# In Intact Islets Interstitial GABA Activates GABA_A_ Receptors That Generate Tonic Currents in α-Cells

**DOI:** 10.1371/journal.pone.0067228

**Published:** 2013-06-24

**Authors:** Yang Jin, Sergiy V. Korol, Zhe Jin, Sebastian Barg, Bryndis Birnir

**Affiliations:** 1 Molecular Physiology and Neuroscience, Department of Neuroscience, Uppsala University, Uppsala, Sweden; 2 Department of Medical Cell Biology, Uppsala University, Uppsala, Sweden; Sackler Medical School, Tel Aviv University, Israel

## Abstract

In the rat islets γ-aminobutyric acid (GABA) is produced by the β-cells and, at least, the α-cells express the GABA_A_ receptors (GABA_A_ channels). In this study, we examined in intact islets if the interstitial GABA activated the GABA_A_ receptors. We used the patch-clamp technique to record whole-cell and single-channel currents and single-cell RT-PCR to identify the cell-type we recorded from, in the intact rat islets. We further identified which GABA_A_ receptor subunits were expressed. We determined the cell-type of 43 cells we recorded from and of these 49%, 28% and 7% were α, β and δ-cells, respectively. In the remaining 16% of the cells, mRNA transcripts of more than one hormone gene were detected. The results show that in rat islets interstitial GABA activates tonic current in the α-cells but not in the β-cells. Seventeen different GABA_A_ receptor subunits are expressed with high expression of α1, α2, α4, α6, β3, γ1, δ, ρ1, ρ2 and ρ3 subunits whereas no expression was detected for α5 or ε subunits. The abundance of the GABA_A_ receptor subunits detected suggests that a number of GABA_A_ receptor subtypes are formed in the islets. The single-channel and tonic currents were enhanced by pentobarbital and inhibited by the GABA_A_ receptor antagonist SR-95531. The single-channel conductance ranged from 24 to 105 pS. Whether the single-channel conductance is related to subtypes of the GABA_A_ receptor or variable interstitial GABA concentrations remains to be determined. Our results reveal that GABA is an extracellular signaling molecule in rat pancreatic islets and reaches concentration levels that activate GABA_A_ receptors on the glucagon-releasing α-cells.

## Introduction

The pancreatic islets consist of four major cell types: the glucagon-secreting α-cells, the insulin-secreting β-cells, the somatostatin-secreting δ-cells and the polypeptide-producing PP-cells. In addition to the hormones, the cells release small molecules that may act in an auto or paracrine manner [1], [2]. Gamma-aminobutyric acid (GABA) is an extracellular signal molecule in the islets [3], [4], [5], [6]. GABA is formed by the enzyme glutamate decarboxylase (GAD) which catalyses the formation of GABA from glutamate and is located both in the cytoplasm and in synaptic-like vesicles [7], [8], [9], [10], [11]. Once released, GABA is thought to act in an auto and paracrine manner on the islet cells to modulate hormone secretion [6], [12], [13], [14], [15], [16], [17].

GABA activates ionotropic GABA_A_ and metabotropic GABA_B_ receptors in the plasma membrane of the islet cells [6], [14], [18]. In the rat islet, only the α-cells express the GABA_A_ receptors (GABA_A_ channels) [19], whereas in human pancreatic islets, the α, β and δ-cells all have GABA_A_ receptors [12], [17]. There are many subtypes of GABA_A_ receptors whereas only one GABA_B_ receptor has been described so far [20]. The GABA_A_ receptors are pentameric. The subunits are grouped into eight families (α1–6, β1–3, γ1–3, δ, ε, θ, π, ρ1–3) and the receptors commonly contain at least 3 different types of subunits: 2 αs, 2 βs and a third subunit-type. The physiological and pharmacological properties of the receptors are determined by the subunit-types that form the GABA_A_ receptors [21].

When GABA binds to the GABA_A_ receptor, a chloride-permeable ion channel is opened. The activation of GABA_A_ channel is best studied in the central nervous system where the receptors evoke phasic (transient) and tonic (long-lasting) inhibition. Phasic activation is mediated by synaptic GABA_A_ receptors and is triggered by the transient, high concentration of GABA (∼mM) released from the presynaptic terminal whereas tonic activation of the extrasynaptic receptors is evoked by the ambient GABA concentration present around the neuron [22]. In the rat β-cells, the vesicular release of GABA coincides with the release of the insulin containing granules when the cell is exposed to high glucose stimulation [11] whereas the non-vesicular release of GABA appears to take place both in high and low glucose concentration [23]. This raises the question of the mode of activation of the GABA_A_ receptors in the pancreatic islet. So far, most of the electrophysiological studies of GABA_A_ receptors in pancreatic islet cells have been conducted on dispersed cells [6] or transfected cells overexpressing GABA_A_ receptors [12], [19]. These studies have, therefore, not resolved the mode of GABA_A_ receptors activation in intact islets. One reason why physiological experiments have predominantly used dispersed cells is related to the difficulty of identifying the cell-types in intact islets. Here we have used the method of single-cell RT-PCR to distinguish the type of cell we recorded from. Our results show, in intact rat pancreatic islets, that interstitial GABA generates tonic currents in the α-cells when the islets are exposed to 20 mM glucose. The tonic current can be enhanced by pentobarbital and inhibited by SR-95531, both drugs specific for GABA_A_ receptors.

## Materials and Methods

### Preparation of Pancreatic Islets

The experiments were carried out on intact rat pancreatic islets isolated from 50–52 days old Wistar rats. Care and use of animals were in accordance with local ethical guidelines and approved by the Uppsala Djurförsöksetiska Nämnd, Sweden (Uppsala’s animal ethics committee). Isolation of pancreatic islets followed a collagenase digestion procedure [24]. Islets were picked by hand with a regular pipette. Isolated islets were cultured for no more than five days in RPMI 1640 medium (Sigma, St. Louis, MO) containing 10 mM glucose, supplemented with 10% (vol/vol) fetal calf serum, benzylpenicillin (100 U/ml), and streptomycin (0.1 mg/ml). The medium was replaced every second day.

### Whole-cell Recording and Current Analysis

To record from rat islets, the intact islet was held by a holding pipette and approached by the recording pipette from the other side [17], [25]. Establishing a GΩ seal on cells was mainly attempted on the surface of the rat islets where the most α-cells are located. After establishing a whole-cell patch-clamp configuration, currents were recorded.

All patch-clamp recordings were performed at room temperature (20–22°C). All drugs used were purchased from Sigma-Aldrich (Steinheim, Germany) or Ascent Scientific (Bristol, UK). Pentobarbital and SR-95531 were dissolved in the extracellular solution. For the voltage-clamped, gap-free recordings, pancreatic islets were perfused in the standard extracellular solution consisting of (mM): 138 NaCl, 5.6 KCl, 2.6 CaCl_2_, 1.2 MgCl_2_, 5 HEPES (pH 7.4 using NaOH) and 20 mM glucose. For the recording of the current-voltage (IV) relationship, 20 mM tetraethyl-ammonium (TEA) chloride replaced 20 mM NaCl in the extracellular solution. The pipette solution consisted of (mM): 125 CsCl, 30 CsOH, 1 MgCl_2_, 10 EGTA, 5 HEPES (pH 7.15 with HCl), and 3 Mg-ATP. The recording pipettes were made from borosilicate glass and had the resistance of 5–8 MΩ when filled with the pipette solution. Recordings were made at −70 or −90 mV. The access resistance was monitored and if it changed by more than 25%, the recording was rejected. Current-voltage (IV) recordings were done in the presence and absence of SR-95531, a GABA_A_ receptor antagonist. The net current activated by the interstitial GABA is calculated by subtractions of current before and after application of SR-95531 (I – I_SR-95531_ = I_GABA_). To record the IV relationship, the cell was held at a holding potential of 0 mV for 200 ms and then stepped to a test potential between –80 and +80 mV in 20 mV increments for 800 ms and then returned to 0 mV for 200 ms before the next test potential was applied.

Patch-clamp recordings were done using an Axopatch 200B amplifier, filtered at 2 kHz, digitized on-line at 10 kHz using an analogue-to-digital converter and analyzed with pClamp (Molecular Devices) and Sigmaplot programs. The amplitude of the tonic currents was measured as the difference in the mean holding current before and after drug application as described by Glykys and Mody [26]. The single-channel recordings were performed in the whole-cell patch-clamp configuration. The amplitude of the currents was measured either from all-point amplitude histograms or from direct measurements of the amplitude of individual currents.

### Harvesting the Cytoplasm

At the end of patch-clamp recordings, the cell’s cytosome was aspirated into the recording pipette by applying a negative pressure to the pipette. Harvesting was interrupted before or as soon as the seal was lost, as described by Lambolez B *et al.* [27]. The content in the pipette (∼5 µl) was then expelled into a PCR tube and frozen immediately on the dry ice. The recording pipettes and the pipette solution were autoclaved and the recording electrode was cleaned with ethanol and *RNAse away* (Molecular Bioproduct, San Diego, CA, USA) before the experiment was started.

### Single-cell RT-PCR

We examined two protocols for RT-PCR identification of gene product: the standard RT-PCR and multiplex RT-PCR. Both protocols worked for islet cell-type discrimination.

For the standard RT-PCR, in the first step, the reverse transcription was performed with Verso™ cDNA synthesis kit (Thermo Scientific, Bremen, Germany) in a final volume of 20 µl. The reverse transcription reaction was incubated at 42°C for 30 min and then at 95°C for 2 min. In the second step, each 3 µl cDNA was individually amplified by specific primer pairs for insulin, glucagon, and somatostatin (Ins, Gcg-2 and Sst-2 in Table 1), by a 40 cycles PCR protocol (95°C, 15 s; 59°C, 30 s; 72°C, 60 s). 4 µl of each individual PCR reaction was examined by electrophoresis on a 1.5% agarose gel stained with SYBR Gold (Invitrogen, Carlsbad, CA, USA).

**Table 1:** Primers used to amplify cDNAs of rat insulin (Ins), glucagon (Gcg-1, Gcg-2), somatostatin (Sst-1, Sst-2), GABA_A_ receptor subunits and the reference gene β-actin (ACTB).

Gene	Primer	Product size (bp)
Ins	F: CAGCAAGCAGGTCATTGTTCC	195
	R: TTCACGACGGGACTTGGGTG	
Gcg-1	F: CGGAGGAGAACGCCAGATCA	133
	R: CGGCGGGAGTCCAGGTATTT	
Gcg-2	F: GCTGGCAGCATGCCCCTCAAG	328
	R: CGGCCTTTCACCAGCCAAGCA	
Sst-1	F: CGGGAAACAGGAACTGGCCAAG	82
	R: AGGCTCCAGGGCATCGTTCT	
Sst-2	F: GTCCTGCCGTCTCCAGTGCG	140
	R: CCAGTTCCTGTTTCCCGGTGGC	
α1	F: TGCCCATGCCTGCCCACTAAAA	511
	R: GCCATCCCACGCATACCCTCTCT	
α2	F: AAAAGAGGATGGGCTTGGGA	550
	R: ACGGGATGTTTTCTGCCTGTAT	
α3	F: TGGGAAGAACAAATCTGTGGAAGTAGC	466
	R: CATCTCCAGGGCCTCTGGTACCTT	
α4	F: TTTAAACGAATCCCCAGGACAGAA	389
	R: TGCCATTTCTCATAATTCTAA	
α5	F: TTATTCTTACTGGGAATGGACAATGG	350
	R: TTAAACCGCAGCCTTTCATCTTTC	
α6	F: CAAGCTCAACTTGAAGATGAAGG	450
	R: TCCATCCATAGGGAAGTTAACC	
β1	F: ACAGCTCCAATGAACCCAGCAA	521
	R: TGCTCCCTCTCCTCCATTCCA	
β2	F: GGAGTGACAAAGATTGAGCTTCCT	564
	R: GTCTCCAAGTCCCATTACTGCTTC	
β3	F: CCGTCTGGTCTCCAGGAATGTTGTC	411
	R: CGATCATTCTTGGCCTTGGCTGT	
γ1	F: CAATAAAGGAAAAACCACCAG	374
	R: TGATTATATTGGACTAAGCCAGA	
γ2	F: GTGAAGACAACTTCTGGTGACTATGTGGT	460
	R: CATATTCTTCATCCCTCTCTTGAAGGTG	
γ3	F: CTGCTGCTTCTCCTCTGCCTGTTC	433
	R: GGTTGGGTGTGGTGATCCAGTGA	
ρ1	F: TGGACAGCAGCTACAGTCACGG	209
	R: AAGCAGCTGGGAAAATGATC	
ρ2	F: CAAGAAGCCACATTCTTCCA	133
	R: TTCTGGAAGATATAGAGTCC	
ρ3	F: GGTGTGAGCGCCTCTATGC	70
	R: GGGAGCTGACCCACATGTACA	
δ	F: GCTCCTGCTGCTCTGCAC	100
	R: GTTGGGGAGCCAGGATATTT	
ε	F: GGCGTGAACAACAAAACTGA	154
	R: AGAAGGAGACCCAGGAGAGC	
θ	F: AGATTTTGGACAGGGTGCTG	82
	R: GATACTGACACAGGCACAGGAG	
π	F: GTGTAGAAGCCCTAGTGTGACC	351
	R: CCACCAATGAATTGACGCAAACCC	
ACTB	F: CCTGGCACCCAGCACAAT	144
	R: GGGCCGGACTCGTCATACT	

The two steps of multiplex PCR were performed essentially as described by Ruano D *et al*. In the first step, cytosome, RT/Platinum Taq Mix (0.4 µl, Invitrogen) and 0.67 picomole of each of the primer pairs (Ins, Gcg-1 and Sst-1 in Table 1) were added to reaction mix supplied by the manufacturer (final volume 20 µl). The set of primers were verified in jPCR program [28] to avoid dimmers or possible interactions among different primers. The reverse transcription reaction was conducted at 55°C for 30 min followed directly by 40 PCR cycles (95°C, 15 s; 59°C, 30 s; 72°C, 60 s). In the second step, 0.4 µl of the first PCR product was then used as template. Each cDNA was individually amplified using gene specific primer pair by performing 40 PCR cycles (same as described above). 4 µl of each individual PCR reaction was then electrophoresised on a 1.5% agarose gel stained with SYBR Green (Invitrogen, Carlsbad, CA).

### Quantitative PCR (RT-qPCR)

Gene expression profiling of the GABA_A_ receptor subunits in rat islets was done on total RNA from isolated rat pancreatic islets by RT-qPCR as previously described [29]. All primers are listed in Table 1.

### Statistics

Data are presented as mean ± standard error of mean (SEM). Differences in expression levels were analyzed by Student’s t-test or non-parametric Mann–Whitney test. In all tests, p<0.05 was considered statistically significant. All statistical tests were performed using SPSS version 18.0 software (SPSS, Chicago, IL, USA) or Sigma Plot version 12 (Systat Software, San Jose, CA, USA).

## Results

### Identification of Cell-types in Intact Rat Islets by Single-cell RT-PCR

**Figure 1:**
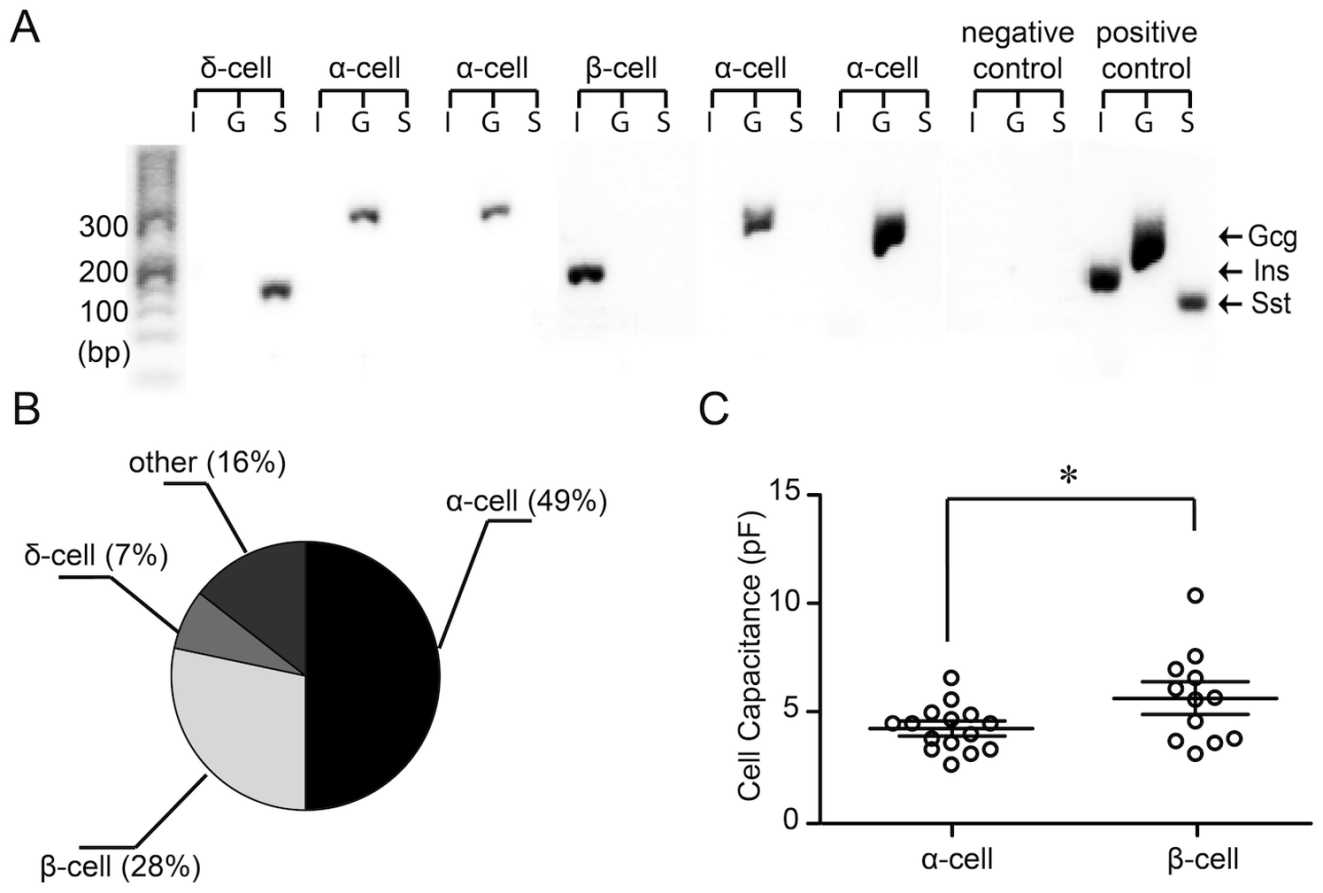
Identification of cell-types in rat pancreatic islets. **A**. Hormone gene expression in single cells. Every three lanes on the gel contain RT-PCR products amplified with insulin (I), glucagon (G) and somatostatin (S) primers: Ins (195 bp), Gcg2 (328 bp) and Sst2 (140 bp), respectively (from left to right). The cell-type was identified according to which product had been detected. Positive controls were purified rat islets RNA samples and the negative control was water. **B**. The pie diagram illustrates the proportion of different cell-types discriminated by single-cell RT-PCR. **C.** Cell capacitance of α-cells (n = 18) and β-cells (n = 12). *P<0.05.

We examined the gene expression of insulin (Ins), glucagon (Gcg) or somatostatin (Sst) in islet cells using single-cell RT-PCR analysis. We did not examine the expression of the pancreatic polypepetide produced by PP cells in this study. The PP-cells constitute less than 5% of the total islet cells [30], [31]. At the end of whole-cell recordings from cells in intact islets the cytosome containing the mRNAs was collected and used for analysis. Fig. 1A shows representative results from 6 different cells. The cytosome of each cell was incubated with three different primer pairs aimed at amplifying the different transcripts encoding the three hormones. The rat islet total RNA was used as positive control and water or the pipette solution as negative controls. Based on amplification of glucagon, insulin or somatostatin gene products, the cells in Fig. 1A were identified as α-cells (n = 4), one as a β-cell and another was a δ-cell.

We collected cytosomes from 95 cells and detected expression of a hormone in 43 of those cells. The majority of the cells were α-cells expressing glucagon (21 cells, 49%, Fig. 1B). 12 cells were β-cells (28%, Fig. 1B) expressing insulin and another 3 cells were δ-cells (7%, Fig. 1B) expressing somatostatin. The remaining 7 cells (16%) expressed somatostatin together with either insulin or glucagon (Fig. 1B). The cell surface area, which is proportional to the cell membrane capacitance, has been used to aid in determining the cell-type. Electrophysiological studies on single islet cells have shown that the β-cells have, on the average, significantly larger cell capacitance than the α-cells [25], [32], [33]. We, therefore, examined the relationship between the cell capacitance and the cell-type we had determined by using RT-PCR. In agreement with previous report Fig. 1C shows that the average cell capacitance was significantly larger for the β-cells (5.6±0.6 pF, n = 12) as compared to the α-cells (4.2±0.3 pF, n = 18). However, since there is a significant overlap between the capacitance of α-cells and β-cells, single-cell RT-PCR further aids in distinguishing the cell-type and may be particularly important in intact islets where cells may be electrically connected.

### mRNA Expression of GABA_A_ Receptor Subunits in Rat Islets

**Figure 2:**
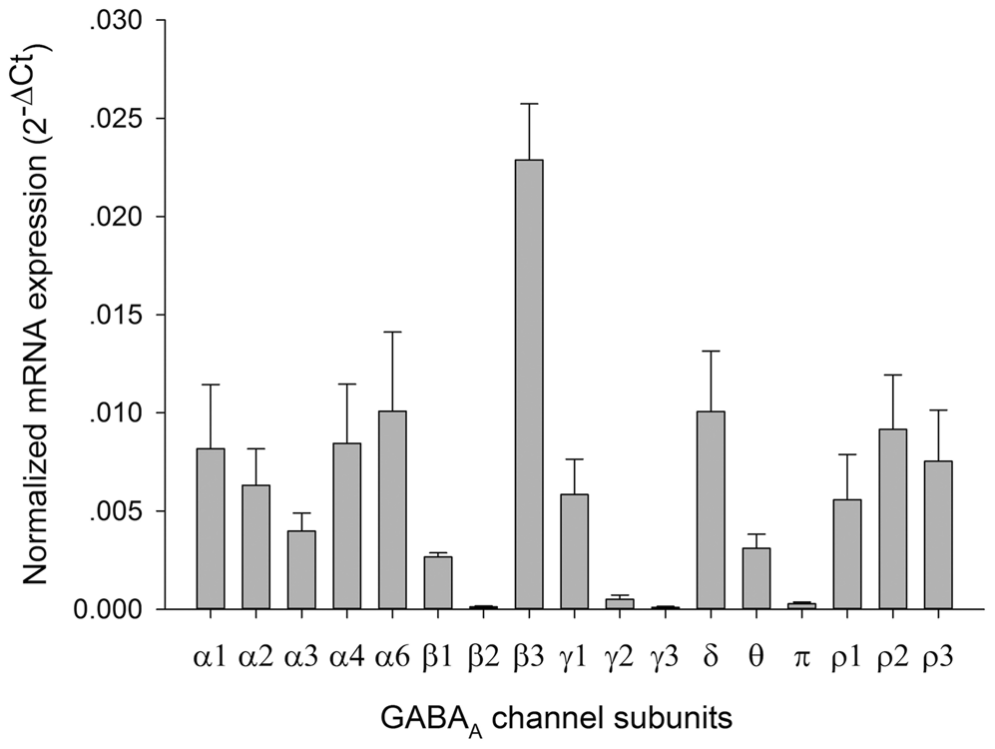
Gene expression of GABA_A_ receptor subunits in rat pancreatic islets. Seventeen GABA_A_ receptor subunits were detected with the most prominent expression level for α1, α2, α4, α6, β3, γ1, δ and ρ1, ρ2, ρ3 subunits. The data is the average expression in islets from 6 rats.

In order to determine which subtypes of GABA_A_ receptors might be expressed in the rat islets, we examined expression of the 19 GABA_A_ receptor subunit isoforms by RT-qPCR and the results are shown in Fig. 2. Seventeen subunits were detected in the islets (α1, α2, α3, α4, α6, β1–3, γ1, γ2, γ3, δ, θ, π and ρ1–3) with the β3 subunit most prominently expressed and relatively high expression of the α1, α2, α4, α6, γ1, δ and ρ subunits. Interestingly, the α5 and the ε subunits were not expressed.

### Interstitial GABA Activates GABA_A_ Receptors and Generates Tonic Currents in Intact Rat Islets

**Figure 3:**
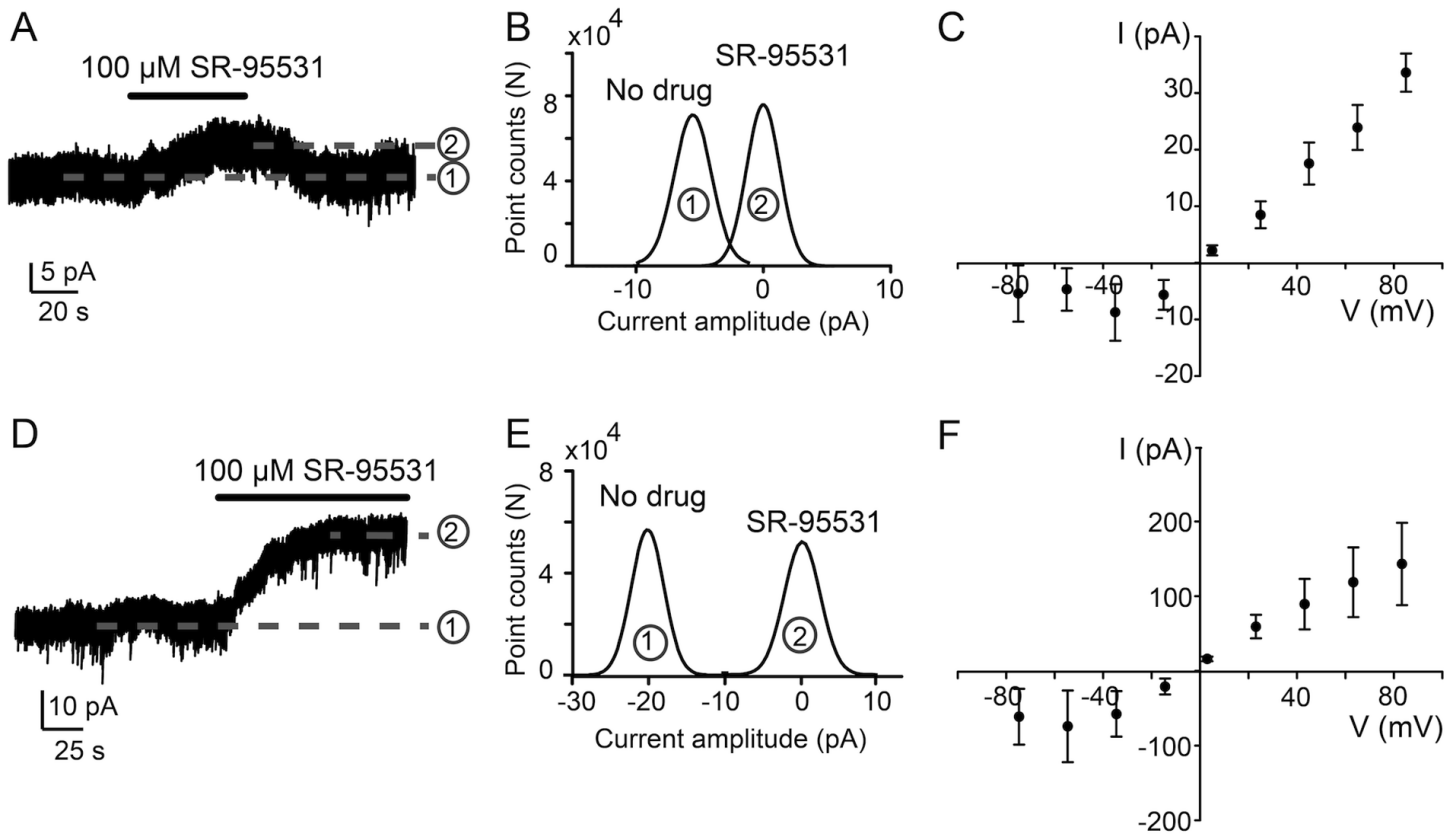
Interstitial GABA activates tonic GABA_A_ receptor currents in rat pancreatic islets. **A.** A representative trace showing GABA-activated tonic current, recorded from a cell in an intact rat islet (Vp = −90 mV). The current was activated by interstitial GABA and inhibited by application of SR-95531, causing a clear outward shift in the holding current (line 1 to line 2). **B**. Gaussian fits to all-points histogram derived from 20 s current recordings in (A) before (1) and after (2) application of SR-95531. SR-95531 insensitive current was subtracted for analysis. The difference between the peaks of the Gaussian fits denotes the mean tonic current (5.3 pA). **C.** Whole-cell current-voltage (IV) relationship of GABA-activated current in rat islet cells (n = 3). The current activated by the interstitial GABA (I – I_SR-95531_ = I_GABA_) is plotted. The IV relation shows outward rectification. **D.** A representative current showing GABA-activated tonic current (21 pA, Vp = −90 mV) that is significantly larger than the current shown in (A). **E**. Gaussian fits to all-points histogram derived from 20 s current recordings in (D) before (1) and after (2) application of SR-95531. SR-95331 insensitive current was subtracted for analysis. **F**. Whole-cell IV relationship of GABA-activated current in rat islet cells (n = 3). The IV relation shows outward rectification.

To-date the interstitial GABA concentration in rat islets has not been determined but we know that GABA is released by both vesicular and nonvesicular release processes [11], [23] and in high concentrations of glucose, at least the vesicular release is stimulated [11]. We, therefore, used 20 mM glucose to evoke GABA release from the β-cells and thereby maximize the interstitial GABA concentration in the islets during our experiments. The highest concentration is expected to be around the β-cells release sites from where it then diffuses to the rest of the islet. We examined if the interstitial GABA activated GABA_A_ receptors in the islet cells and the results are shown in Fig. 3. Although no GABA was added experimentally, whole-cell currents were reduced by SR-95531, a GABA_A_ receptor specific antagonist. SR-95531 reduced the baseline current but increased again when SR-95531 was withdrawn. The difference in the whole-cell current level in the presence and absence of SR-95531 revealed the level of the GABA-activated tonic current in the cell (Fig. 3A, B) which was 5.3 pA in the cell shown (Vp = −90 mV, Fig. 3B). In eight different cells the average tonic current level was 5.2±2.5 pA (n = 8, Vp = −90 mV). The current-voltage (IV) relation of the GABA-activated current was outwardly rectifying (Fig. 3C) demonstrating that more current passes through the GABA_A_ receptors at depolarized as compared to hyperpolarized potentials in rat pancreatic islets. In another four cells the tonic current was significantly larger (Fig. 3D and E, 21 pA, Vp = −90 mV; Fig 3F). Whether the larger current level is due to more GABA_A_ receptors in the plasma membrane or different subpopulations of GABA_A_ receptors expressed in the cells is not known. However, insulin promotes insertion of GABA_A_ receptors into the plasma membrane [34]. As insulin is released in high glucose this might have happened in some of the cells and we captured it in the four cells with the larger currents before the receptor number decreased again in the plasma membrane.

**Figure 4:**
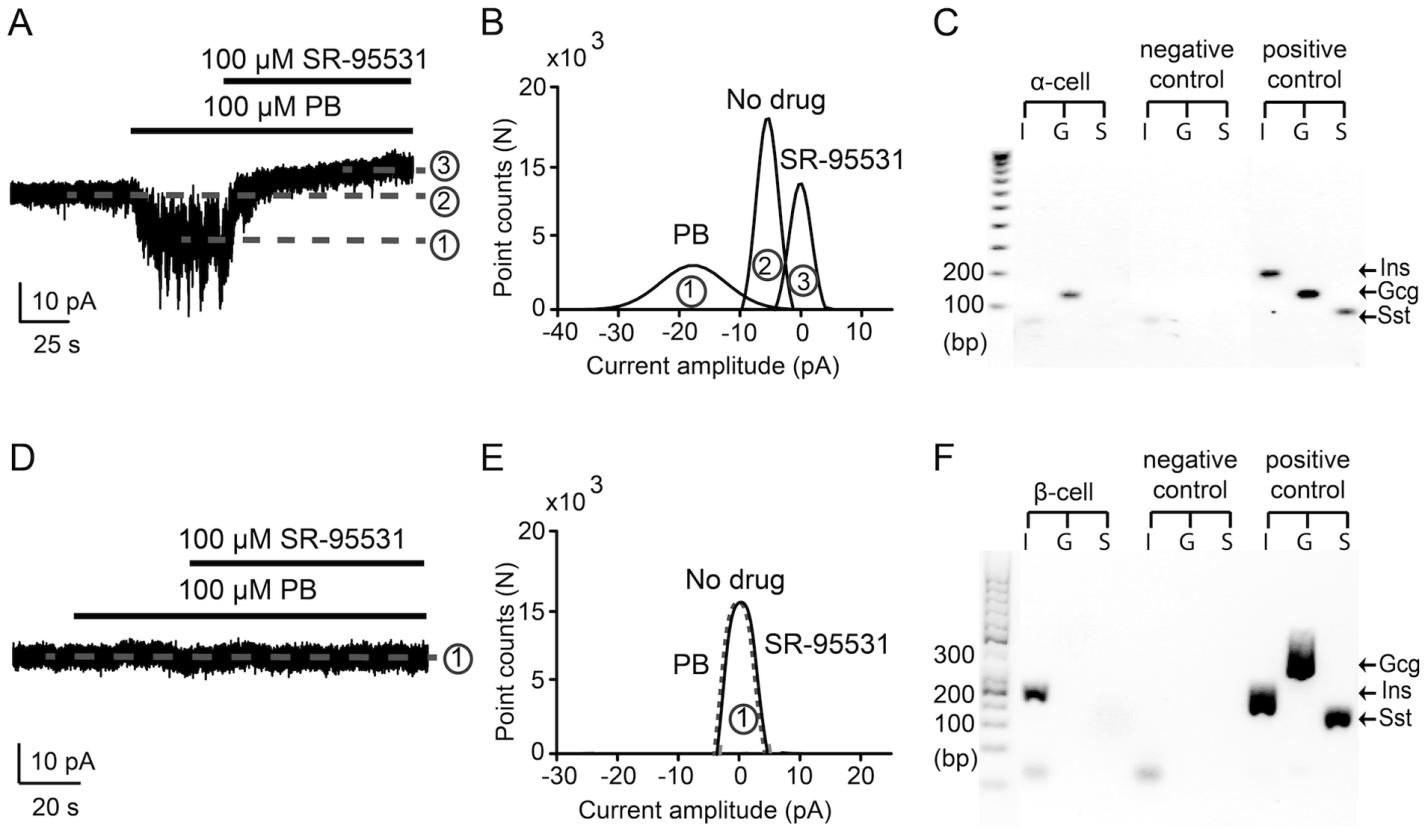
Pentobarbital (PB) enhances interstitial GABA-activated currents in pancreatic islet α-cells. **A.** A representative current trace shows interstitial GABA-activated tonic current (2) that was potentiated by pentobarbital (1) and then inhibited by SR-95531 (3) (Vp = -70 mV). **B.** Gaussian fits to all-points histogram derived from 20 s current recordings in (A) before (2) and after application of (1) pentobarbital and (3) SR-95531 SR-95531 insensitive current was subtracted for analysis. The difference between the peaks of the Gaussian fits shows the evoked (5 pA, (2) – (3)) and the enhanced (17 pA, (1) – (3)) tonic currents. **C.** Electrophoresis image of single-cell RT-PCR products from the cell in (A). Every three lanes on the gel contain amplified RT-PCR products: insulin (primer: Ins), glucagon (primer: Gcg1) or somatostatin (primer: Sst1). The cell-type was identified as an α-cell. Positive controls were purified rat islets RNA samples and the negative control was water. **D**. A representative current recording showing whole-cell current that did not respond to pentobarbital or SR-95531 (Vp = −70 mV). **E.** Gaussian fits to all-points histogram derived from 20 s current recordings in (D) before and after application of pentobarbital and SR-95531. SR-95531 insensitive current was subtracted for analysis. **F.** Electrophoresis image of single-cell RT-PCR products from the cell in (D). Every three lanes on the gel contain amplified RT-PCR products: insulin (primer: Ins), glucagon (primer: Gcg2) or somatostatin (primer: Sst2). The cell-type was identified as a β-cell. Positive controls were purified rat islets RNA samples and the negative control was water.

Pentobarbital modulates GABA_A_ receptors resulting in enhanced current response to GABA [21]. We examined if pentobarbital potentiated the tonic GABA-activated currents in cells in the intact islet and the results are shown in Fig. 4A and B. The application of 100 µM pentobarbital enhanced the tonic current by 12.4 pA (171 pS, Vp = –70 mV) and significantly increased the current baseline noise. Similar results were obtained in another 3 cells where pentobarbital increased the GABA_A_ receptors conductance (111.8±21.6 pS, n = 4). The cytosome of the cell from which the current was recorded (Fig. 4A) was harvested and the cell shown to be an α-cell (Fig. 4C). GABA-activated currents were recorded in another 7 α-cells. For the twelve identified β-cells, successful whole-cell current recordings were obtained in seven of these cells but in none of them was GABA-evoked current detected (n = 7, Fig. 4D, E, F).

### Single-channel Recordings of GABA_A_ Receptor Currents in Intact Rat Pancreatic Islets

**Figure 5:**
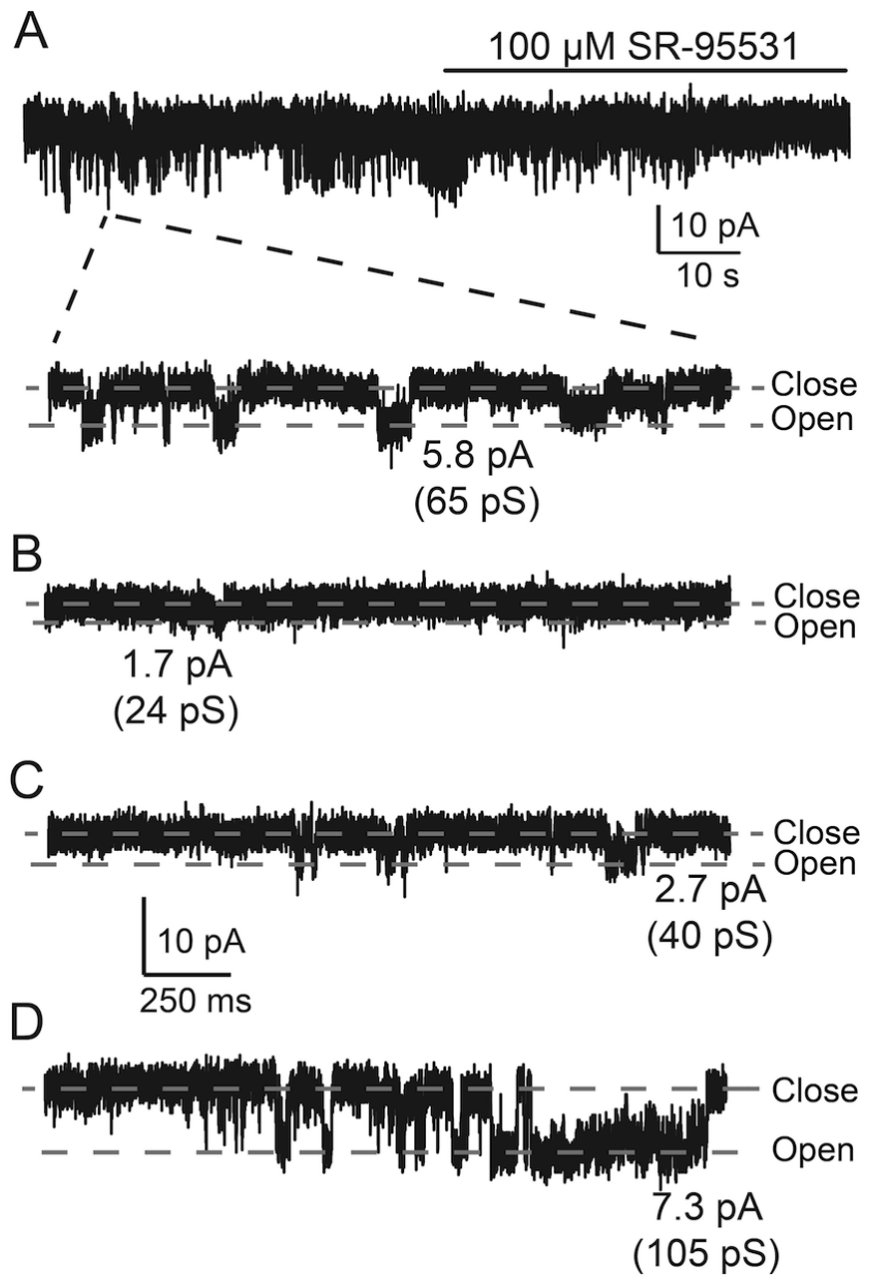
Interstitial GABA activates single-channel currents in intact islets. Single-channel currents that were later inhibited by 100 µM SR-95531 were recorded in four different cells (**A**, **B**, **C** and **D**) in intact rat islets. **A.** A representative current trace (top trace, slow time scale, s) showing GABA-activated single-channel currents and inhibition by 100 µM SR-95531. The solid line shows the time when the extracellular solution containing SR-95531 was perfused through the recording chamber. The broken lines indicate from where in the recording the current trace on the faster time scale (ms) was obtained. The glucose concentration was 20 mM and the holding potential was −90 mV (**A**), −70 mV (**B–D**). The most prominent single-channel conductance in the cells in A**,** B**,** C and D were 65 pS, 24 pS, 40 pS and 105 pS, respectively. For all four cells, the currents were recorded in the whole-cell patch-clamp configuration from cells in intact islets and were activated by interstitial GABA and inhibited by application of SR-95531 (100 µM).

Under our experimental conditions, the membrane resistance of the cells in the whole-cell patch-clamp configuration was often in the GΩ range indicating that very few channels were open in the cell membrane. In this situation, the whole-cell can be regarded as an unusually large outside-out membrane patch and ideally suited for single-channel recordings. In Fig. 5, four representative current recordings are shown where the interstitial-GABA activated single-channel currents in cells in intact islets. The most prominent single-channel conductances were 65 pS (Fig. 5A, Vp = −90 mV), 24 pS (Fig. 5B, Vp = −70 mV), 40 pS (Fig. 5C, Vp = −70 mV) and 105 pS (Fig. 5D, Vp = −70 mV). When the islets were perfused with 100 µM SR-95531, the single-channel currents shown in Fig. 5 were inhibited. A current trace on a slow time scale (s, Fig. 5A, the top current trace) demonstrates the SR-95531 inhibition of the channels in one of the cells. The average conductance of native rat GABA_A_ receptors recorded at the holding potential of −70 or −90 mV was 42±7 pS (n = 13).

### GABA-activated Single-channel Currents Decrease Gradually in 100 µM SR-95531

**Figure 6:**
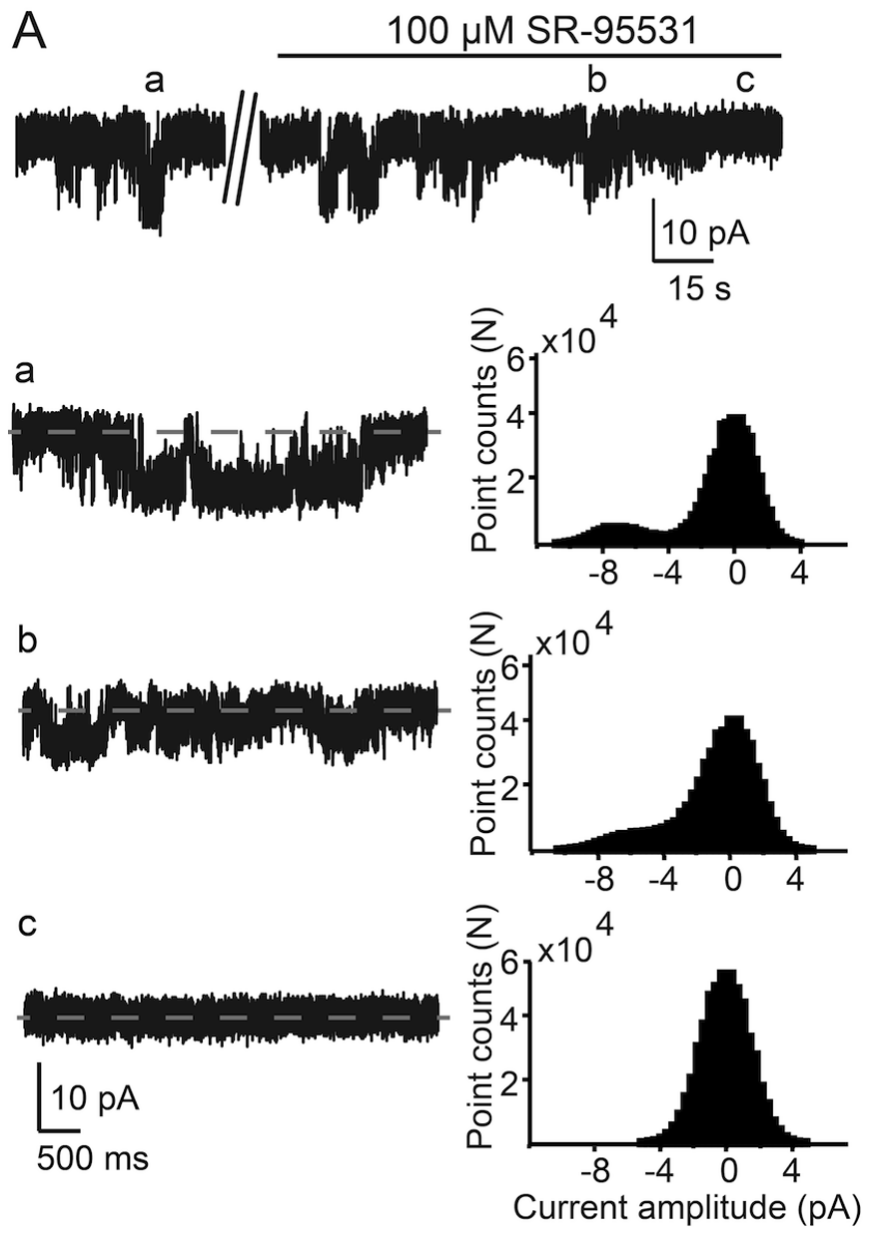
SR-95531 gradually inhibits the GABA-activated single-channel currents. **A.** A current trace at a slow time scale (s) showing GABA-activated single-channel currents at the beginning of an experiment and then inhibition by 100 µM SR9553 (Vp = −90 mV). The solid line shows the time when the SR-95531 was applied to the islet. The currents on a faster time scale is shown in **a, b** and **c** and the all-points histograms (20 s) include the corresponding current trace. The channel activity was completely abolished by 100 µM SR 95531 after 90 s perfusion.

The current recordings in Fig. 6 (Vp = −90 mV) show that SR-95531 gradually decreases the channel conductance in intact islets. The all-points histograms are from 20 s current episodes and include the corresponding current trace. Before exposure to SR-95331, the maximum channel conductance was 105 pS (Fig 6A and Aa) but after about 1 min in 100 µM SR-95531, the conductance had decreased to 35 pS (Fig 6A and Ab) and 30 s later the channel was no longer detectable (Fig 6A and Ac).

### Pentobarbital Enhances the GABA-activated Single-channel Currents

**Figure 7:**
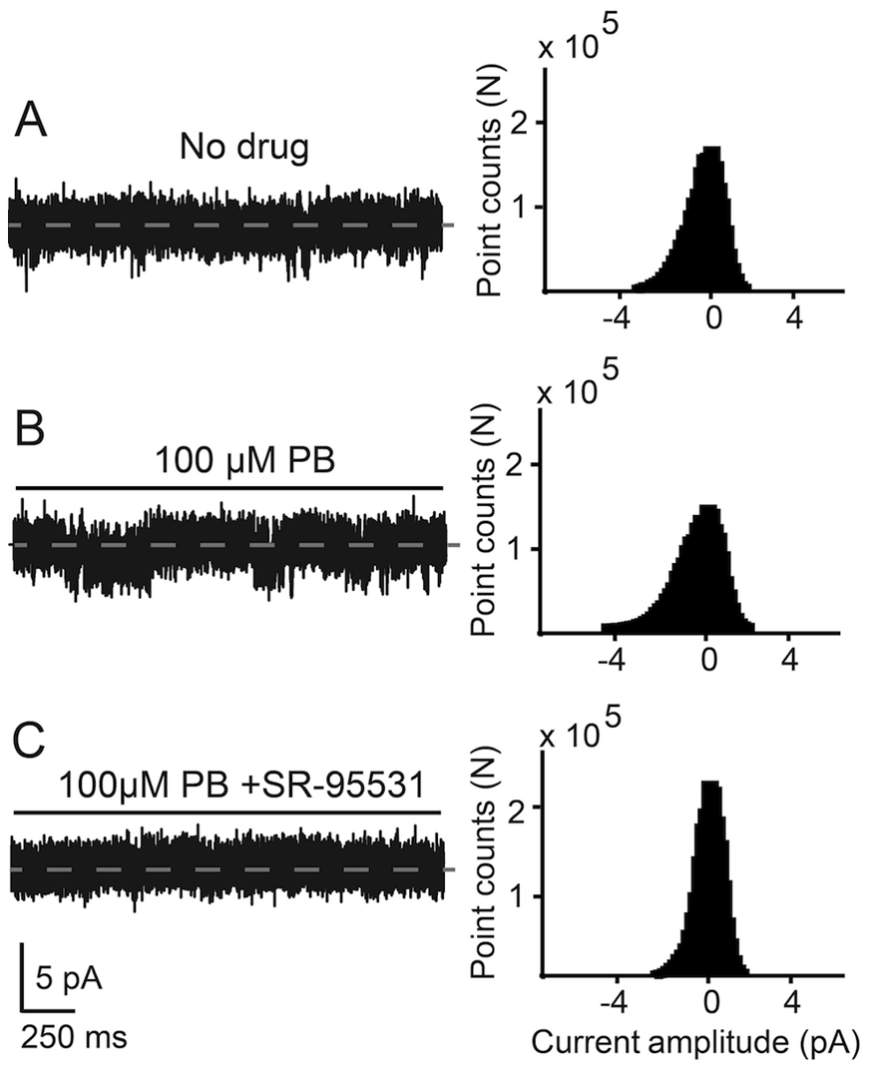
Pentobarbital (PB) enhances the GABA-activated single-channel current. Single-channel currents recorded from the same cell is shown in **A**, **B** and **C** (Vp = −70 mV) and the all-points histograms (20 s) include the corresponding current trace. The solid line shows the time when the extracellular solution containing drug was perfused through the recording chamber. 100 µM pentobarbital potentiated and 100 µM SR-95531 inhibited the GABA-activated currents.

An example of the effect of 100 µM pentobarbital on GABA-activated single-channels in the intact islets is shown in Fig. 7. Before the application of pentobarbital, the receptors had an average current amplitude of 1.6 pA (23 pS, Vp = −70 mV, Fig. 7A) and a low open probability as shown by the all-points histogram (15 s) to the right of the current trace. Following exposure to pentobarbital (Fig. 7B), both the single-channel current and the open probability increased as manifested in channel current of 2.7 pA (38 pS, Vp = −70 mV) and the skewed all-points histogram. Similar results were obtained in another four experiments where the pentobarbital-enhanced average conductance was 38±6 pS. 100 µM SR-95531 inhibited the GABA-activated, pentobarbital-enhanced currents (Fig 7C).

## Discussion

Our results demonstrate that GABA acts as a paracrine signaling molecule in the intact rat pancreatic islets. The results show that the interstitial GABA concentrations activate GABA_A_ receptors generating tonic currents that are only present in the glucagon-releasing α-cells in rat islets. The results are consistent with the observation that antagonists of GABA_A_ receptors inhibit glucagon secretion in both rat and human islets [17], [19]. Identification of the types of cells the recordings were made from in the intact islets was done by the single-cell RT-PCR. The single-channel conductance of GABA_A_ receptors ranged from 24 to 105 pS. Both the whole-cell and the single-channel currents were enhanced by pentobarbital and inhibited by GABA_A_ receptors antagonist SR-95531.

The success rate of identifying the cells we recorded from, in the intact islets, was about 45% using the single-cell RT-PCR technique. About 50% of the cells were α-cells although they represent only 20% of all the cells in a pancreatic islet. This result is related to the way the experiments were executed as we most commonly record from cells on the surface of the islets. Most of the surface cells in rat islets are non-β cells [25], [30]. We, nevertheless, were also able to record from the β-cells, about 30% of the identified cells, which make up the core of the rat islet and represent 60–70% of the islet cells [25], [30]. The β-cells were, on the average, significantly larger than the α-cells as determined by the cell-capacitance and the results are consistent with what has been reported previously in mouse islets [25], [33]. We did, nevertheless, observe significant scatter in size for both the α- and β-cells highlighting the need for an alternative method, the single-cell RT-PCR, to determine the cell-type in islets.

Although the presence of the GABA_A_ receptors in pancreatic islets has been known since the late 1980s [6], only recently was it shown that the interstitial GABA in the islets activates the native GABA_A_ receptors [17]. GABA_A_ receptors can be supersensitive to GABA and respond when the GABA concentration is as low as pM to nM [35], [36]. In this study, no external GABA was added but tonic, GABA-generated currents were recorded. The GABA must, therefore, have originated from cells within the islets. Furthermore, since the GABA-activated current was enhanced by pentobarbital, the interstitial GABA concentration must have been sub-saturating [17], [35], [36].

GABA_A_ receptors normally contain two αs, two βs plus a third type of subunit in the receptor complex [21]. In the rat islets, we detected 17 of the total 19 possible GABA_A_ receptor subunit genes. The highest expression level was detected for the β3 subunit and a very low expression level for β2, γ2, γ3 and π. The α5 and ε subunits were not expressed. Clearly a number of different GABA_A_ receptor subtypes can be expressed in the rat islets and is in accord with earlier reports that have examined the expression of the subunits in rodents [19], [34]. Interestingly, in human islets, both the α5 and π subunits are prominently expressed [12], [17]. As the subunit composition determines both the physiological and the pharmacological properties of the receptors, it can be expected that although GABA signaling takes place both in human and rodent islets, the pharmacological profile of the response will vary somewhat between species [21], [37].

The type of α subunits influences the GABA affinity of the receptors [21]. A number of different GABA_A_ receptor α subunits are expressed in the islets potentially forming receptors that differ in their sensitivities to GABA and other drugs [21], [35], [36], [37]. The whole-cell IV curves showed outward rectification. GABA_A_ receptors have been shown to have either linear or outwardly rectifying IV, both at the single-channel level and at the whole-cell current level [36], [38], [39], [40]. At the single channel level, the outward rectification is the property of the ion channel molecule whereas at the whole-cell current level it can be either due to intrinsic properties of the ion channels or that the open probability of the channels is lower at hyperpolarized potentials as compared to depolarized potentials. The tonic conductance varied significantly in magnitude, which might be related to different density of the GABA_A_ receptors expressed in the cell plasma membrane, the concentration of interstitial GABA or the subtypes of GABA_A_ receptors in the plasma membrane. At the single-channel level, GABA_A_ receptors were gradually blocked by SR-95531 and potentiated by pentobarbital. Whether the single-channel conductances we determined in this study are related to different GABA_A_ receptor subtypes or activation of a specific GABA_A_ receptor-type by different GABA concentrations remains to be examined.

In the central nervous system, GABA-activated tonic current has an important physiological function in regulating overall excitability of neural networks [22]. Similarly, it is possible that tonic activation of GABA_A_ receptors in pancreatic islets have a role not only in suppressing the membrane potential but even synchronizing the population of the α-cells, in addition, to coordinate their activities with that of the β-cells. GABA is known to exert inhibitory effect on glucagon release [6], [17], [34]. Clearly, extracellular GABA signaling in pancreatic islets is of physiological and potentially pharmacological significance for glucose homeostasis. Our results demonstrate that GABA signaling can be studied in identified cells in intact islets by combining the single-cell RT-PCR method with patch-clamp recordings.
